# Sporadic Burkitt Lymphoma First Presenting as Painful Gingival Swellings and Tooth Hypermobility: A Life-Saving Referral

**DOI:** 10.3390/dj13010006

**Published:** 2024-12-25

**Authors:** Erofili Papadopoulou, Maria Kouri, Dimitrios Velonis, Anastasia Andreou, Maria Georgaki, Spyridon Damaskos, Evangelia Piperi, Konstantina Delli, Ioannis K. Karoussis, Antonia Vlachou, Georgia Avgerinou, Antonis Kattamis, Nikolaos G. Nikitakis

**Affiliations:** 1 Department of Oral Medicine & Pathology and Hospital Dentistry, School of Dentistry, National and Kapodistrian University of Athens (NKUA), Greece 2 Thivon Street, 11527 Athens, Greece; kourimari@yahoo.gr (M.K.); dimitrios.velonis@gmail.com (D.V.); andreouanast@gmail.com (A.A.); margeor@dent.uoa.gr (M.G.); liapiperi@dent.uoa.gr (E.P.); nnikitakis1@yahoo.com (N.G.N.); 2 Department of Oral Diagnosis and Radiology, School of Dentistry, National and Kapodistrian University of Athens (NKUA), 11527 Athens, Greece; sdamask@dent.uoa.gr; 3 Department of Oral Diseases and Oral and Maxillofacial Surgery, Faculty of Medical Sciences, Hanzeplein 1, HPC BB70, Postbus 30001, 9700 RB Groningen, The Netherlands; k.delli@umcg.nl; 4 Department of Periodontology, School of Dentistry, National and Kapodistrian University of Athens (NKUA), 11527 Athens, Greece; ikaroussis@dent.uoa.gr; 5 Division of Pediatric Hematology-Oncology, First Department of Pediatrics, School of Medicine, National and Kapodistrian University of Athens (NKUA), 11527 Athens, Greece; avlachou@live.com (A.V.); g.avgerinou@yahoo.gr (G.A.); ankatt@med.uoa.gr (A.K.)

**Keywords:** Burkitt lymphoma, sporadic, first manifestation, jaws, gingival swelling, teeth hypermobility

## Abstract

**Background:** Burkitt lymphoma (BL) is an aggressive non-Hodgkin lymphoma (NHL), subdivided into endemic, sporadic, and immunodeficiency-associated forms. While jaw lesions are common in endemic BL, they are infrequent in sporadic cases, only rarely constituting the first manifestation of the disease. The aim of this study is to present a rare pediatric case of sporadic BL first manifesting as gingival swellings and tooth hypermobility and provide a review of all the published sporadic BL case reports as the first sign of disease. **Case report:** An 11-year-old Caucasian female was referred for the evaluation of hypermobility of posterior lower teeth, associated with painful gingival swellings of 20 days duration. Clinical examination revealed right facial asymmetry and bilateral prominent swellings of the posterior lower gingiva. A panoramic radiograph revealed ill-defined radiolucent lesions in the posterior mandible bilaterally. On computed tomography, soft-tissue masses were identified along the mandibular ramus extending into the maxillary sinus bilaterally. The histopathologic and immunohistochemical analyses of the lesions led to a diagnosis of Burkitt lymphoma (BL). The patient underwent a full staging work-up, revealing bone marrow involvement and widespread disease. A multi-chemotherapy regimen was initiated with the regression of oral lesions and symptoms within a few weeks and complete disease remission after nine chemotherapy cycles. The patient remains free of disease 11 years later. **Conclusions:** This case underscores the critical importance of the timely diagnosis and life-saving referral of rapidly growing jaw lesions, which may represent the first sign of an underlying lymphoreticular malignancy with aggressive course, such as BL.

## 1. Introduction

Non-Hodgkin lymphomas (NHLs) account for about 4% of all malignant diseases in children younger than 15 years old [1]. Burkitt lymphoma (BL) is an aggressive non-Hodgkin B cell lymphoma with the potential to involve multiple organ systems, characterized by the translocation of the *MYC* oncogene [2].

BL was first described in 1958 by Denis Burkitt as a mandibular malignancy in African children [3,4]. Over time, the recognition of cases worldwide indicated its universal relevance. It is subdivided into three clinical variants with distinct epidemiologic, immunologic, and molecular/cytogenetic characteristics: endemic, sporadic, and immunodeficiency-associated [5].

The most common variant is endemic BL which accounts for 30 to 50% of all childhood cancers in equatorial Africa, with an estimated incidence of 3 to 6 cases per 100,000 children per year [2,6]. It has a peak incidence between ages 6–8 and a male predominance. It appears more frequently in the jaws and Epstein–Barr virus (EBV) is detected in around 95% of the cases [3,7].

Sporadic BL is a rare malignancy among Western populations, with a reported annual incidence of 2–3 cases per million. Still, it accounts for approximately 30–50% of childhood lymphomas, but less than 1% of all NHL in adults [2,8,9]. Sporadic BL appears more frequently in the abdominal region and EBV is detected in about 10–30% of the cases [3].

Immunodeficiency-associated BL is mostly associated with HIV infection, accounting for 20–40% of HIV-associated NHLs [10]. In fact, BL is thought to appear early in HIV infection, before CD4+ cell numbers drop [3,11,12]. This type of BL can also be seen in immunosuppressed allograft recipients and patients with congenital immunodeficiency. It more frequently develops in lymph nodes, bone marrow, and central nervous system [2,5].

BL shows a broad spectrum of clinical manifestations, usually with multi-organ involvement. The African or endemic variant often involves the jaws and other facial bones, while head and neck manifestations in sporadic BL are considered rare [2].

The aim of the present manuscript is to describe a case of sporadic BL with jaw involvement, manifesting with painful gingival swellings and tooth hypermobility as the first signs of the underlying widespread disease. Moreover, the findings of all the published case reports of sporadic BL with oral involvement as first occurrence are summarized in order to enhance the diagnosis of similar cases.

## 2. Case Presentation

An 11-year-old female was referred for the evaluation of bilateral painful swellings on the gingiva of the lower premolars and molars, associated with tooth hypermobility. The painful symptoms had started 3 weeks before and were initially attributed to erupting second permanent mandibular molars; however, they were progressively worsening, accompanied by difficulty in swallowing, general malaise, and weight loss. Further, a noticeable, right facial asymmetry had also developed over the last 3 days. The patient had been examined by a general dentist and an ENT specialist, who had prescribed antibiotic treatment (amoxicillin for 7 days), analgesics, and antiseptic mouthwashes, without improvement. Following consultation with a periodontist, the child was referred to a specialized Oral Medicine Clinic.

The patient’s medical history was unremarkable and a blood test, performed on the same day, was within normal limits. No fever or lymphadenopathy was present.

**Figure 1:**
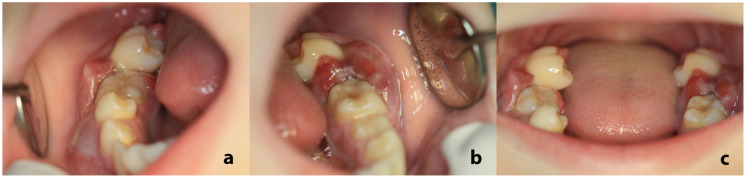
Intraoral examination: prominent gingival swelling adjacent to the premolars and molars of the right (**a**) and left (**b**) mandible; the gingiva were erythematous and focally ulcerated. The aforementioned teeth, especially the second molars, were hypermobile, displaced, and partially extruded (**c**).

On clinical examination, an extraoral facial swelling along the right posterior mandible was noticeable. Intraorally, bilateral prominent swellings were identified, bilaterally affecting the mandibular premolar and molar gingiva, which were erythematous and focally ulcerated (Figure 1a,b). The aforementioned teeth were extremely hypermobile, some of them displaced and partially extruded (Figure 1c).

**Figure 2:**
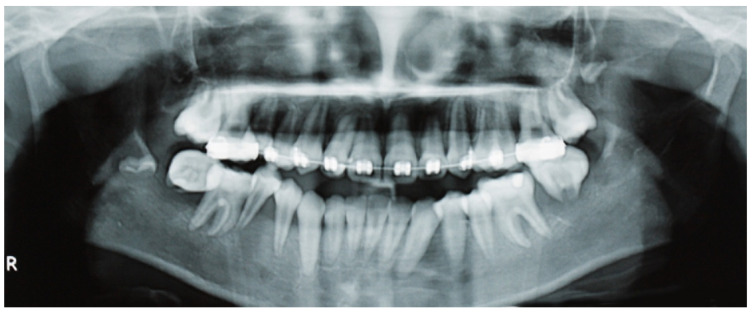
Panoramic radiography shows ill-defined radiolucencies in the posterior mandible bilaterally, affecting the adjacent permanent teeth that resemble a ‘floating teeth’ appearance. In this particular focal trough, the osteolysis appears to be more diffuse in the right mandible, extending into the premolar region and also causing the displacement of the sperm of the third molar.

**Figure 3:**
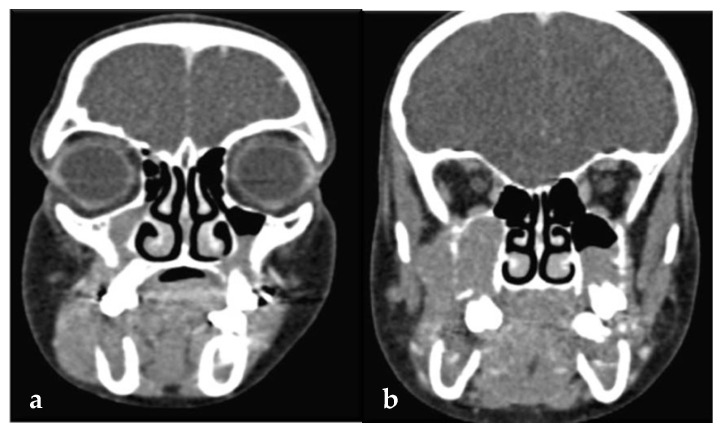
(**a**,**b**): Computed tomography showing hypodense (soft tissue) masses along and distal to the mandibular ramus extending into the maxillary sinus, bilaterally.

A panoramic radiograph revealed ill-defined radiolucencies in the posterior mandible bilaterally, related to the adjacent permanent teeth, which also showed a ‘floating teeth’ appearance (Figure 2). Moreover, computed tomography showed hypodense (soft tissue) masses along and distal to the mandibular ramus extending upwards into the maxillary sinuses, bilaterally (with dimensions of 1.5 × 3.0 × 7.0 cm and 1.7 × 1.2 × 3.5 cm on the right and left side, respectively), as well as the posterior wall of the right orbit; also, there was a perforation of the right mandibular cortex along the second molar tooth (Figure 3 a,b). Based on the clinical and imaging findings, the differential diagnosis mainly included hematologic neoplastic conditions, such as lymphomas, leukemias, and Langerhans cell disease; the possibility of sarcomas, such as rhabdomyosarcoma or Ewing sarcoma, was also considered, especially considering the patient’s age.

**Figure 4:**
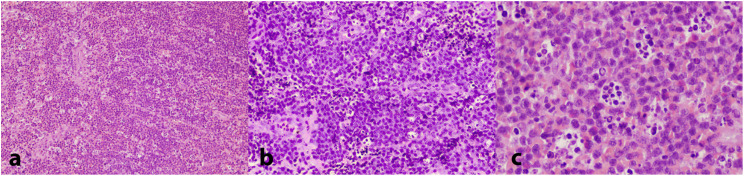
Histopathologic examination of the biopsy specimen showing a dense infiltrate of lymphocytes of medium size with minimum cytoplasm and round nuclei exhibiting a significant mitotic rate; a ‘starry sky’ pattern, due to abundant indispersed macrophages, is apparent. (H&E stain, (**a**): 25×; (**b**): 100×; (**c**): 200×).

**Figure 5:**
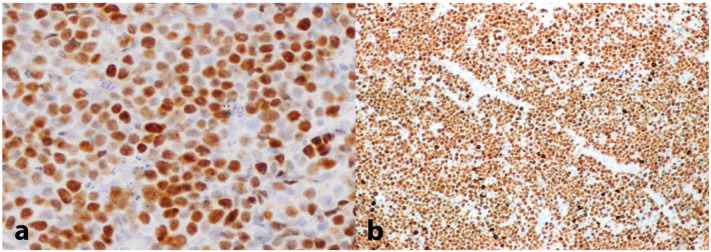
Immunohistochemical evaluation showing (**a**) intense positivity for B-myc (200×) and (**b**) a Ki-67 proliferation index approximating 100% (25×).

Taking into account the aggressive presentation, a partial biopsy of the affected gingiva in the area of the right second mandibular molar was deemed as an imminent need and was performed on the day of the first visit. Histopathologic findings revealed a dense proliferation of lymphocytes of medium size with minimum cytoplasm and round nuclei with multiple nucleoli, exhibiting a significant mitotic rate and dispersed apoptotic bodies; in addition, abundant indispersed macrophages, generating a ‘starry sky’ appearance, were noticed (Figure 4a–c). An immunohistochemical analysis revealed that the tumor cells were positive for CD20, CD79a, CD10, Bcl-6, and Bmyc (Figure 5a); in contrast, they were negative for Bcl-2, Tdt, Cyclin-D1, MUM1, and myeloperoxidase, while CD3 and CD5 were positive only in a small number of reactive T lymphocytes. In addition, there was kappa light chain restriction, while the Ki-67 cell proliferation index was positive in almost all the neoplastic cells (Figure 5b). Based on the histopathologic and immunohistochemical findings, a final diagnosis of BL, sporadic type, was rendered.

**Figure 6:**
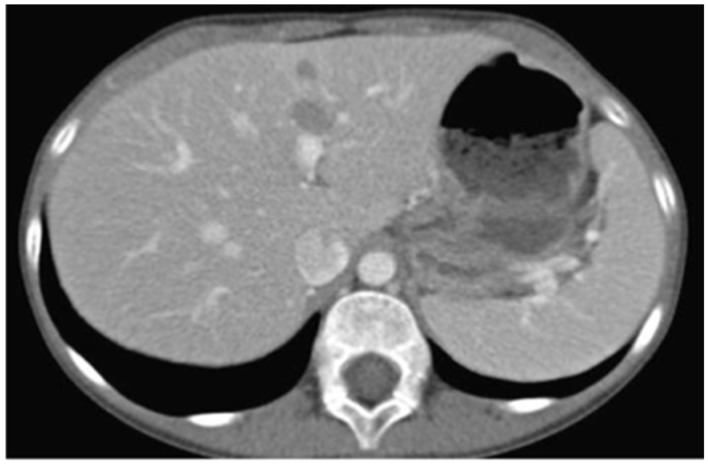
Abdominal CT showing diffuse tumor infiltration of the liver.

The patient was promptly referred to the Department of Pediatric Oncology of the Children’s Hospital for complete work-up and staging. Full body CT and PET/CT scanning revealed multiple lesions in the skeleton (including the skull, vertebrae, ribs, humerus, femur, and pelvis), liver (Figure 6), and pelvic lymph nodes. Bone marrow aspiration revealed infiltration (15–20%) by neoplastic lymphocytes. The disease was classified as stage IV without CNS involvement.

The patient stratified into Group B according to the staging of the disease and received treatment according to the FAB-LMB 96 protocol. Treatment included nine cycles of chemotherapy, with 21-day intervals. Due to inadequate response after the fourth cycle of chemotherapy, the patient was switched to Group C, Arm C1, and the patient achieved Complete Remission at the end of the treatment.

**Figure 7:**
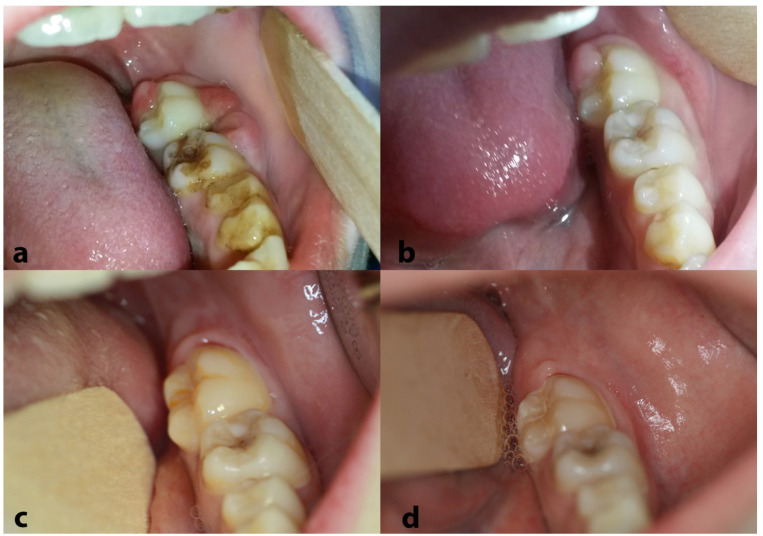
Clinical appearance of the lesions in the left posterior mandible after 10 days (**a**) and 15 days (**b**) from the biopsy; please note the significant regression just a few days following the beginning of chemotherapy. No relapses were noticed during the long-term follow-up [representative clinical views at 9 months (**c**) and 32 months (**d**) from the diagnosis].

Regarding oral lesions and symptoms, rapid regression was noted right after the initiation of the first chemotherapy cycle (Figure 7a,b). At the end of the chemotherapy, a PET/CT scan confirmed that the patient was free of disease; regular and thorough follow-up, including PET/CT, magnetic resonance imaging (MRI) of the brain and visceral skull, ultrasonography (U/S) of upper and lower abdomen and/or chest X-ray, and appropriate blood tests, confirmed sustained complete remission. Similarly, the oral cavity remained free of lesions during the long-term follow-up (Figure 7c,d). Eleven years later, the patient remains free of disease and totally healthy.

## 3. Discussion

BL derives from germinal center B cells and its three subtypes are thought to arise from these cells at different stages of their development [2]. BLs are composed of monomorphic CD19+; CD20+, commonly IgM+ B cells, exhibiting basophilic cytoplasm; numerous mitotic figures; and a Ki67 score of more than 95% [3]. The tumor cells exhibit a high degree of apoptosis, and a ‘starry sky’ pattern is seen at low magnification in H&E-stained slides [13]. The ‘sky’ is formed by round basophilic cells without intercellular stroma and the ‘stars’ correspond to numerous, scattered benign histiocytes [14].

Although the histopathologic appearance is the same, the three BL subtypes vary in clinical features and, especially, age predilection and area of distribution. The endemic form develops in Africa and occurs in early childhood, while the sporadic form shows no geographic predilection and occurs more commonly in children and adolescents. The endemic form mostly involves the jaws, causing tooth mobility and jaw expansion, while the sporadic form is not common in the jaws [15,16,17]. Immunodeficiency-associated BL is mainly seen in HIV patients and only uncommonly affects the jaws [3,5,11,12].

When the jaws are involved, the mandible, especially the posterior part, is more frequently affected [18]; mandibular swelling is the most common clinical feature, as was in our case. Another common clinical symptom is mental nerve neuropathy, also known as ‘numb chin syndrome’, in which the patient complains about chin and lower lip paresthesia [19,20]. Pain and tooth mobility are also frequent, as in our case, and may often lead the clinician to a misdiagnosis of odontogenic infection, causing unnecessary dental interventions, delays in diagnosis and treatment, and worse prognosis [15]. Other clinical signs of jaw involvement include the loosening and extrusion of the molar teeth (primary and permanent), premature shedding of primary molars and eruption of permanent molars, gingival enlargement, maxillary sinus obliteration, and facial asymmetry [21], some of these features being prominent in the present case too.

In cases of jaw involvement, a panoramic radiograph is a helpful initial approach in order to exclude signs of odontogenic infection/inflammation, which is the most prevalent cause of facial swelling in children [18]. The most common radiographic features are non-specific osteolytic lesions with ill-defined borders and, perhaps, an image of ‘floating-in-air’ teeth, like in our case. Other radiographic features of BL may include tooth displacement, lamina dura loss, and periodontal ligament widening [19]. Ultrasonography may be helpful in case of palpable masses in the neck. CT and MRI are valuable to identify bone resorption and adjacent structure involvement and to define the precise extent of the tumor. The appropriate imaging of the chest, abdomen, and pelvis and, especially, PET-CT are useful for tumor staging, as well as for the evaluation of tumor response to therapy [18].

Diagnosis is confirmed via the biopsy of a specific disease site or lymph node. In addition to the aforementioned histopathologic features, the expression of markers typical of germinal center B cells is characteristic [14]. BL expresses monotypic surface IgM, CD19, CD20, CD79a, PAX5, CD43, the plasma cell antigen CD38, and the germinal center antigens CD10 and BCL6, while the Ki-67 proliferative fraction is >95% [14]. Additional tests include bone marrow biopsy, spinal fluid examination, kidney and liver function assessment, and testing for HIV disease [22].

Microscopic differential diagnosis includes hematologic diseases, especially other types of B cell lymphomas, such as diffuse large B cell lymphoma (DLBCL), unclassifiable BL/DLBCL that has an extremely poor prognosis, lymphoblastic lymphoma, blastoid mantle cell lymphoma, and leukemia [6,14].

EBV was identified in 1964 in endemic (African) type BL. The prevalence of EBV infection varies greatly in the different BL subtypes and there are still unresolved questions concerning the contribution of EBV to BL oncogenesis. Endemic BL cases are considered at least 95% EBV-associated, with supportive evidence, including the presence of EBV-DNA clonally integrated into tumor cells and epidemiologic associations with serum EBV antibodies, indicating that EBV infection precedes the malignant transformation [3,23]. On the other hand, only one-third of non-endemic BL is EBV-positive [5]. The variable EBV association in the three BL variants has prompted many to contemplate the possibility of the virus being a passenger in the neoplastic process and not the initiating factor. It is believed that EBV, analogous to malaria, leads to polyclonal B cell activation and permits the poorly controlled proliferation of EBV-positive B cells, further leading to a greater risk of *c-myc* rearrangement and lymphomagenesis [24]. More recent studies propose a hit-and-run mechanism for the EBV-negative BL. It is suggested that EBV plays an initiating role in oncogenesis, but the viral genome is lost [3].

In all variants, irrespective of EBV status, the constitutive activation of the *c-myc* (*c-MYC*) oncogene (human genes are to be written in capital italicized letters I think) is clearly the oncogenic key factor through its translocation, involving the long arm of chromosome 8 and the Ig heavy chain gene on chromosome 14 (>85% of cases) or the Ig light chain genes, [25] leading to the expression of MYC. The *MYC/Ig* translocation may not be highly detected by common cytogenetics compared to fluorescence in situ hybridization or polymerase chain reaction that increases the chance of positive verification [26]. Interestingly, the position of chromosomal breakpoint relative to the human *c-MYC* gene differs between endemic and sporadic BL, suggesting different genetic mechanisms involved [27].

The stage of non-Hodgkin lymphoma is determined according to the number of groups of lymph nodes affected and their distribution in the body, as well as the involvement of other organs, such as bone marrow or liver. The system that is still used for BL classification is the Ann Arbor or the St Jude/Murphy staging system, extending from I to IV [28]. Frequently, patients are presented with late disease stages III–IV (70% of the cases). Similarly, in the present case, the patient was categorized as stage III after a thorough work-up.

BL is fatal if left untreated. Prognosis depends on the extent of the disease, the patient’s age, and the timing of diagnosis, described as excellent for the early/moderate stages (survival rate reaching 97–98%), while in the advanced stages (III or IV) it drops to 87.3% [29]. The features associated with an adverse outcome include older age, advanced stage, poor performance status, bulky disease, high lactate dehydrogenase (LDH), and central nervous system (CNS) or bone marrow involvement [30]. In fact, in a recent study, four clinical factors were identified as independently prognostic for patients’ outcomes: age ≥ 40 years, LDH > 3× normal, Eastern Cooperative Oncology Group Performance Status (ECOG PS) ≥ 2, and CNS involvement [31].

The current treatment is chemotherapy with multi-agent regimes, including doxorubicin, vincristine, alkylators, and etoposide, for either short or longer duration [2]. More recently, monoclonal antibodies have been used as adjuvant therapy in BL. In this respect, the anti-CD20 monoclonal antibody rituximab has been used in combination with chemotherapy for the treatment of BL especially for advanced stages, where it has shown improvement in survival by over 95% [2,29]. Radiotherapy is reserved for overt CNS disease resistant to chemotherapy and is reported to be useful in certain emergencies, such as airway obstruction. Hematopoietic stem cell transplantation remains experimental in adults and could be an option in younger patients with refractory/resistant or relapsed disease [32]. The surgical management of BL is limited to emergency cases, necessitating the removal of parts of the intestine that are blocked, bleeding, or have ruptured [33]. Due to the rapidly growing knowledge about the molecular biology of the disease, novel treatment options are in early development, including epigenetic regulators like histone deacetylase inhibitors and DNA methyltransferases inhibitors, as well as small peptide nucleic acids to target oncogenes [32].

Close monitoring during treatment and surveillance following its completion are mandatory; follow-up is recommended every 2–3 months with thorough clinical examination, imaging studies, and blood investigation [2]. The frequency of surveillance decreases over the years since more relapses occur in the first year after treatment completion [29].

In the case presented here, the most clinically significant aspect from a diagnostic standpoint was the fact that the first manifestations were in the mandible, presenting with clinical signs and symptoms that mimicked common innocuous dental inflammatory conditions. Following initial empirical attempts to alleviate the patient’s symptoms on the presumption that they experienced secondary tooth eruption and associated pericoronitis, appropriate referral to a specialized clinic and immediate diagnostic work-up, both imaging and microscopic, was virtually life-saving; it ensured establishing the correct diagnosis and initiating the appropriate chemotherapy in a timely fashion in the face of a very aggressive and rapidly progressing neoplasm.

Accordingly, we performed a review of all the published sporadic BL cases in the English language literature, focusing on well-documented reports with oral and maxillofacial involvement as the first manifestation of the disease. The cases were divided depending on the age of the patients, i.e., children (0–17 years) or adults (≥18 years). The results, including age, sex, presenting sign/symptom, clinical characteristics, imaging features, and outcome, are presented in Table 1 [34,35,36,37,38,39,40,41,42,43,44,45,46,47,48,49,50,51,52,53,54,55,56,57,58,59,60,61,62,63,64,65,66,67] and Table 2 [19,21,43,61,68,69,70,71,72,73,74,75,76,77,78,79,80,81,82,83,84,85,86,87,88,89,90,91,92,93,94,95,96,97,98], respectively. In summary, there were 44 [15,18,34,35,36,37,38,39,40,41,42,43,44,45,46,47,48,49,50,51,52,53,54,55,56,57,58,59,60,61,62,63,64,65,66,67] cases of sporadic BL in children, including our case. The age distribution ranged between 3 and 16 years, with a mean age of 7.9 years, while the male to female ratio was 2.1:1. In adults, there were 38 cases of sporadic BL; the age distribution ranged between 18 and 84 years, with a mean age of 43 years, while the male to female ratio was 2.8:1.

In children, the most common presenting oral and maxillofacial signs and symptoms, corresponding to the first manifestations of the disease, were swelling (in 68.2% of the cases, more frequently localized in the mandible in 34.1%) and pain (34.1%, frequently presenting as toothache); tooth hypermobility or loosening was a chief complaint in 18.2% of the cases, while paresthesia was rarely reported. In adults, swelling (52.6%, more frequently in the mandible in 28.9%) and pain (42.1%, sometimes in the form of toothache) were also common, similar to children; on the other hand, paresthesia, especially numbness of the chin and/or lower lip, was much more common (50%), while tooth hypermobility was only rarely a chief complaint (5.3%) in adults. It should be mentioned that in many cases, lesions were misdiagnosed as dentoalveolar infections and clinicians proceeded with root canal treatment or the extraction of teeth in the lesional area, followed by an antibiotic regimen, with only the deterioration of the symptoms and progression of the disease.

The main finding of clinical examination, in both children and adults, included a swelling and/or mass (84.1% and 71.1%, respectively); th edetection of palpable lymph nodes was comparable among children and adults (27.3% and 21.1%, respectively). In contrast, tooth mobility and/or displacement were much more common in children (61.4% vs. 18.4% in adults), while neurological signs (paresthesia/hypoesthesia/anesthesia) were more frequent in adults (28.9% vs. 4.5% in children). In several cases, other remote sites of involvement were noticed following further investigation at the time of diagnosis.

Various imaging modalities, including panoramic and periapical X-rays, CBCT, CT, MRI, and PET/CT, aided in the diagnosis and allowed the identification of the exact localization of the involved areas and the extent of the disease into adjacent structures. In most cases, single or multiple lytic/destructive bone lesions (93.2% in children and 47.4% in adults) and/or space-occupying lesions (29.5% in children and 18.4% in adults) were noticed. Other imaging findings related to tooth structures included the loss of lamina dura (29.5% in children and 13.2% in adults), teeth displacement (25% in children, but not reported in adults), root resorption (18.2% in children and 7.9% in adults), ‘floating-in-air’ teeth appearance (15.9% in children and 2.6% in adults) and periodontal ligament widening (2.3% in children and 5.3% in adults). Noteworthy is that the overall incidence of the aforementioned imaging findings was almost universally more common in children compared to adults, although the use of different imaging modalities does not allow a direct comparison.

On follow-up (1–192 months for children and 1–60 months for adults), patients were reported to remain free of disease in 50% of children (between 4 and 192 months, mean 59 months) and in 57.9% of adults (between 3 and 60 months, mean 23.2 months). A fatal outcome was reported in 29.5% of children (in 3–10 months following diagnosis) and 26.3% (in 1–11 months following diagnosis) of adults.

## 4. Conclusions

BL is an aggressive B cell lymphoma, but promptly diagnosed, it rapidly responds to treatment and exhibits a satisfactory long-term prognosis. Jaw lesions demonstrating rapid osseous destruction and aggressive clinical course, especially in the absence of an obvious odontogenic etiology, may constitute the early signs of a more serious condition. The timely detection and appropriate referral of such a rapidly growing jaw lesion, similar to the case presented here, may be life-saving, since it can lead to prompt diagnosis and treatment, ultimately contributing critically to a favorable outcome. Regular long-term follow-up, including a thorough dental and oral and maxillofacial examination, in order to detect possible relapses is also indispensable.

## Figures and Tables

**Table 1:** Cases of sporadic Burkitt lymphoma in children (0–17 years) with oral and maxillofacial involvement as the first manifestation of the disease.

	Author	Age (Years)	Gender	Presenting Signs/Symptoms (Oral and Maxillofacial Area)	Clinical Examination	Imaging Examination (Periapical/Panoramic/CBCT/CT/MRI/PET)	Follow-Up
1	Stewart and Whitlock 1972 [34]	7 ½	M	Facial swelling for 3 weeks	Tooth mobility and displacement Anomaly in occlusion Marked bulging of the inner–outer aspects of the maxillary alveolar ridge Facial swelling Orbital edema–ptosis	Loss of lamina dura Teeth displaced Mandibular erosion Extensive destruction of maxillary bone Erosion of the lateral wall of the antrum	Died 5 ½ months later
2	Joncas et al., 1977 [35]	5	M	Mandibular swelling for more than 1 month	Ulcer of mandibular gingiva Tooth mobility Mandibular swelling	Mandibular osteolysis	7 years, no recurrence
3	Terill et al., 1977 [36]	12	F	Mandibular swelling Generalized dull toothache	Teeth loosening Irregular attached gingiva and mucosa Intermittent paresthesia of the lower lip Swelling of the mandibular buccal cortex Palpable submandibular lymph nodes	Diffuse mandibular alveolar bone osteolysis	Died 5 months later
4	Moore et al., 1983 [37]	9	M	Tooth mobility and toothache for a few days Orbital swelling, eye proptosis, and diplopia	Tooth mobility Palpable cervical lymph nodes bilaterally	Mandibular osteolysis Loss of lamina dura Dental papillae appeared larger than normal, tended to blend with the surrounding mandibular body with an appearance of coalescence between adjacent papillae Technetium scan showed increased maxillary and mandibular activity	Died 3 months later
5	Boraz 1983 [38]	9	M	Pain on chewing	Tooth mobility Gingival swelling Facial asymmetry Submandibular swelling	Mandibular osteolysis	2 years, no recurrence
6	Zachariades and Papanikolaou 1986 [39]	7	F	Mandibular swelling for 3 weeks	Soft mass on the mandibular molars Bilateral palpable, painless, freely movable lymph nodes	Floating teeth Diffuse mandibular radiolucency	Died 6 months later
7	Kearns et al., 1986 [40]	3	M	Maxillary mass with teeth loosening (and lethargy for 4 weeks)	Distorted right maxilla with the infiltration of a mass into the hard palate Teeth loosening	Involvement of the right maxillary antrum with associated bone destruction	Disease free, unknown duration
8		5	M	Bilateral cervical masses (and low-grade fever) for 7 weeks	NA	Bilateral maxillary and ethmoid sinus opacification with bone destruction Gallium scan confirmed the presence of nasopharyngeal disease	Disease free, unknown duration
9	Alaluusua et al., 1987 [41]	6	M	Mandibular toothache Mandibular/maxillary swelling	Mandibular gingival mass Teeth extrusion Mandibular and orbital swelling Temporomandibular joints dislocated Jaw movement extremely difficult	Teeth displaced Mandibular/maxillary osteolysis Mandibular asymmetry	Died 10 months later
10	Svoboda et al., 1991 [42]	4	M	Tooth mobility Toothache	Tooth mobility Bilateral mandibular/maxillary buccal vestibules swelling Anterior open bite Palpable submandibular, jugulodigastric, and cervical lymph nodes	Loss of lamina dura Premature root resorption Loss of dental follicles around developing teeth Mandibular/maxillary osteolysis Loss of trabecular bone architecture	Died 10 months later
11	Wang et al., 1992 [43]	6	M	Maxillary tooth pain	Bleeding mass on maxillary alveolar ridge	Bone destruction of the nasopharynx extending to the right maxillary sinus cavity, infratemporal fossa, and right orbit	7 years, no recurrence
12		4	F	Mass in the hard palate and right maxillary sinus	NA	NA	16 years, no recurrence
13		5	F	Facial swelling	NA	NA	18 months, no recurrence
14	Ardekian et al., 1996 [44]	16	M	Mandibular vestibular swelling	Mandibular vestibular and gingival swelling Tooth sensitivity	Mandibular osteolysis Loss of lamina dura Periodontal ligament widening Trabecular bone pattern changed	NA
15	Lund et al., 1997 [45]	5	F	Submandibular swelling Painful cheek for 10 days	Tooth mobility, extrusion, and dislocation Buccal and palatal alveolar expansion Fixed cervical and mandibular swelling	Periapical bone rarefaction Pericoronal osteolysis Displaced germs	2 years, no recurrence
16	Alpaslan et al., 1997 [46]	6	F	Intraoral mass for 5 months	Palpable lymph nodes Mandibular swelling Occlusion abnormalities	Germ displacement Mandibular osteolysis	Recurrence (unknown duration)
17	Hanazawa et al., 1998 [47]	10	M	Submandibular and mandibular swelling Toothache (mandible)	Mandibular gingival swelling Tooth mobility and dislocation Trismus Pharyngeal wall enlarged	Teeth floating, displaced Root resorption Loss of lamina dura Mandibular osteolysis Mass extending to sublingual, submandibular, parapharyngeal, and retropharyngeal spaces	2 years, no recurrence
18	Mitsudo et al., 2000 [48]	16	M	Alveolar bone resorption	Tooth mobility Deep periodontal pocket Gingival swelling	Upper/lower alveolar osteolysis Loss of lamina dura	Died 11 weeks later
19	Tsui et al., 2000 [49]	4	M	Mandibular swelling for 4 days	Mandibular swelling Tooth mobility Palpable submandibular lymph nodes	Mandibular osteolysis	Complete resolution 1 month after chemotherapy
20	Liu et al., 2000 [50]	14	M	Chewing pain at the left upper premolar region Tooth mobility for 1 week Mild toothache	Moderate tooth mobility in almost all permanent teeth Buccal alveolar expansion along the mucobuccal folds of the maxilla and mandible with no clear boundary	Loss of lamina dura Periapical bone loss Mandibular/maxillary osteolysis	Died 12 weeks later
21	Durmus et al., 2003 [51]	10	M	Mandibular swelling (on the left) and pain Facial swelling	Mandibular swelling Tooth mobility, displacement, and extrusion Buccal and lingual alveolar mandibular bone expansion Palpable submandibular lymph nodes	Mandibular osteolysis Tooth extrusion	Died 8 months later
22	Comfort et al., 2004 [52]	5	M	Facial swelling for 2 weeks after tooth extraction	Gingival swelling Palpable cervical and submandibular lymph nodes	No bone destruction Facial swelling	Discharged home 3 weeks after admission with an explanation of the ‘anticipated poor’ prognosis to the parents
23	Jan et al., 2005 [53]	13	F	Spontaneous mandibular toothache for 2 weeks Progressively loosening teeth Inability to chew food comfortably	Tooth mobility and supraeruption Open bite, mandibular swelling Buccal alveolar expansion Pale, swollen, spontaneous bleeding gingiva Palpable submandibular lymph nodes	Teeth floating, displaced Alveolar bone resorption Mandibular osteolysis	NA
24	Ugar et al., 2006 [54]	5	M	History of bilateral cervical masses for 7 weeks	NA	Bilateral maxillary and ethmoid sinus opacification with bone destruction Gallium scan confirmed the presence of nasopharyngeal disease	Disease free, unknown duration
25	Patil et al., 2007 [55]	9	F	Mandibular swelling for 15 days	Tooth mobility Gingival bleeding Solitary, well-defined, lobulated, erythematous mass in the mandibular left posterior region Mandibular swelling	Ill-defined radiolucency with the destruction of trabeculae giving a ‘moth-eaten’ appearance Teeth displaced, ‘floating in air’ Buccal and lingual mandibular cortical plate expansion—Mandibular osteolysis	Disease remission, the patient was asked to report every 2 months for evaluation
26	Freitas et al., 2008 [56]	7	M	Mandibular swelling	Mandibular swelling Mass in the mandibular vestibule	Tooth germs dislocated Mandibular osteolysis Ruptured mandibular cortical layer	7 years, no recurrence
27	Pereira et al., 2010 [57]	4	M	Toothache-like pain for 1 month Mandibular swelling	Mandibular swelling Facial asymmetry	Tooth displaced Mandibular osteolysis	11 months, no recurrence
28	Valenzuela-Salas et al., 2010 [58]	5	M	Painful swelling of maxillary buccal vestibule for 1 month Submandibular adenopathy	NA	Maxillary osteolysis Maxillary sinus opacification Soft mass affecting the right maxillary sinus, orbital floor, and lateral nasal wall	12 years, no recurrence
29	Vasudevan et al., 2011 [59]	13	M	Gingival bleeding Chewing difficulty Facial swelling for 2 months	Mandibular swelling Tooth mobility Palpable lymph nodes	Mandibular osteolysis	Died (unknown duration)
30	Chbicheb et al., 2012 [60]	13	F	Intraoral swelling	Mandibular/maxillary gingival swelling	Mandibular/maxillary osteolysis	2 years, no recurrence
31	Rebelo-Pontes et al., 2014 [61]	9	F	Exophytic mass and pain in the mandible	Painful exophytic swelling in the mandible, tooth mobility	Alveolar bone destruction with root resorption and loss of lamina dura	Died of disease
32		8	M	Facial swelling and pain	Painful mandibular and maxillary swellings, tooth mobility	Alveolar bone destruction with root resorption and the loss of lamina dura	3 years, no recurrence
33		3	M	Bilateral facial swelling and pain	Bilateral exophytic masses in the mandible and maxilla	Alveolar bone destruction with root resorption and the loss of lamina dura	4 months, no recurrence
34		5	M	Cutaneous ulceration, facial asymmetry	Maxillary swelling, tooth mobility	Alveolar bone destruction with root resorption and the loss of lamina dura	Died of disease
35	Cabras et al., 2018 [62]	15	F	Dull pain on the permanent maxillary right second molar	Overall swelling of the gingiva surrounding the permanent maxillary right second molar, extending to the right palatal mucosa Lip hypoesthesia	Panoramic radiograph: no pathologic features CT: solid tissue with high uptake in the right maxilla, with osteolysis and the disruption of the floor of the right maxillary sinus	3 years, no recurrence
36	Cho et al., 2018 [15]	6	M	Hypermobility of the maxillary right first molar	Painless gingival and mucosal swelling in both posterior maxilla and mandible Right mandibular first molar was tilted lingually	Periapical X-ray: severe alveolar bone resorption around the maxillary right 1st molar, with uncompleted root development Panoramic X-ray: the loss of lamina dura around all four first molars CT: tumorous lesions occupying both maxillary sinuses, causing partial resorption of the sinus wall	20 months, no recurrence
37	Yilmaz et al., 2021 [63]	13	F	Mandibular and maxillary hypermobile teeth, pain and numbness of right mandibular region	Swelling of the gingiva around right mandibular and maxillary premolars and molars and mobility of these teeth Facial asymmetry Enlarged lymph nodes	Panoramic X-ray: alveolar bone resorption adjacent to the maxillary and mandibular canines, premolars, and molars with the appearance of floating teeth Periapical X-ray: several root resorptions, punch hole on the maxillary alveolar bone	NA
38	De Coninck et al., 2020 [18]	7	F	Persistent and painful unilateral swelling of the mandible	Intraoral mass in the left mandible interfering with the occlusion	Osteolytic lesions in the lower left quadrant of the mandible Absence of the thin circumferential cortical layer around the left lower second molar Mass in the left masseter muscle region, fixed to the ascending mandibular ramus with osteolysis of the bone	Regression of disease
39	Kulczyk et al., 2021 [64]	11	M	“Inflammation” in the region of the left lower premolar	Hypermobility of the left lower premolars and enlargement and erythema of the vestibular and lingual gingiva with pain	Root resorption of the left lower premolar, rarefaction with the loss of trabecular bone pattern, and the destruction of buccal and thinning of lingual cortical plates in the left mandible (area of the left lower canine, premolar, and first molar), ‘floating-in-air’ appearance	5 years, no recurrence
40		8	M	“Trauma” of the front upper central incisors	Hypermobility of the left upper incisor, swelling and petechiae of the upper lip, dome-shaped enlargement of the palate extending from the midline to the palatal aspect of the left upper first molar	Soft-tissue mass in the area of the hard palate and extending into the maxillary sinus and nasal cavity A blurred pattern of alveolar bone in the region of the left upper molars	7 years, no recurrence
41	Riaz et al., 2021 [65]	3	M	Rapidly enlarging swelling on the right lower side of the face	Extraorally: firm swelling on the right side of the face extending from the cheek bone up to the lower border of the mandible Intraorally: mass on the right posterior mandible, erythematous, with lobulated, irregular surface	Mixed radiolucent radiopaque osteolytic lesion causing the destruction of cortical bone Loss of the lamina dura of the right lower primary first and second molars	Died of disease
42	De Freitas Filho et al., 2021 [66]	5	M	Bilateral mandibular swelling, trismus, pain on the left side of the face, and headache	Ulceration in the left alveolar ridge, swelling with color change in the right alveolar ridge, and tooth mobility of the left lower primary second molar	Solid tissue invading both sides of the maxilla and the maxillary sinus and partially the floor of orbitals	3 years, no recurrence
43	Chait et al., 2024 [67]	7	M	Upper gingival swelling, 1 month following a dental extraction	Gingival swelling with mobility and the displacement of the teeth on the right maxilla	Osteolysis of the maxilla with tooth displacement, tumor occupying the maxillary, and sphenoidal sinuses with left temporal endocranial extension	1 month, complete regression of the lesions
44	Papadopoulou et al., 2024 (present case)	11	F	Painful swellings of the gingiva of the lower molars, tooth hypermobility, and facial asymmetry	Bilateral gingival swellings (erythematous and focally ulcerated) adjacent to mandibular premolars and molars that were hypermobile, displaced, and partially extruded Facial swelling along the right posterior mandible	Panoramic radiograph: ill-defined radiolucencies in the posterior mandible bilaterally, ‘floating-in-air’ teeth appearance CT: hypodense masses along and distal to the mandibular ramus extending upwards into the maxillary sinuses bilaterally, and the posterior wall of the right orbit; perforation of the right mandibular cortex	11 years, no recurrence

**Table 2:** Cases of sporadic Burkitt lymphoma in adults (≥18 years) with oral and maxillofacial involvement as the first manifestation of the disease.

	Author	Age (Years)	Gender	Presenting Sign/Symptom (Oral and Maxillofacial Area)	Clinical Examination	Imaging Examination (Periapical/Panoramic/CBCT/CT/MRI/PET)	Follow-Up
1	Baden and Carter 1987 [68]	18	M	Severe toothache leading to extraction Submandibular swelling	Palpable cervical lymph node Swelling of submandibular triangle Mass protruding from extraction wound at the site of the mandibular third molar	Loss of lamina dura around most of the posterior mandibular teeth Osteolysis of the retromolar triangle (cancellous spaces irregularly enlarged)	Died 1 ½ month later
2	Wang et al., 1992 [43]	22	M	Diplopia, left-sided ptosis, and facial numbness	Diplopia, left lateral rectus muscle weakness, left-sided ptosis, and facial numbness. Later: bilateral ptosis and numbness over the entire distribution of the ophthalmic and maxillary divisions of the left trigeminal nerve	Massive tumor in the ethmoid sinuses, extended into the sphenoid sinus, right orbit, and olfactory foramen	Died shortly after diagnosis
3	Lynch and Harris 1994 [69]	41	F	Chin pain Mandibular swelling Horizontal diplopia	Chin numbness and pain Palpable preauricular and cervical lymph nodes Swelling of the right mandible	Mandibular osteolytic lesion with indistinct margins	Response to induction chemotherapy Died of a herpesvirus infection 50 days after bone marrow transplantation
4	Yoskovitch et al., 2000 [70]	76	M	Enlarging mass on the right dorsal surface of tongue for 5 months Worsening dysphagia and odynophagia	Firm, nontender submucosal mass of the right dorsal aspect of the tongue posteriorly	NA	18 months, no recurrence
5	Landesberg et al., 2001 [21]	28	M	Paresthesia and anesthesia of the lower lip and chin area	Tooth mobility 5 days after the initial examination expansion of the mandibular buccal cortex Anesthesia on the 3rd division of trigeminal nerve	No pathologic features on panoramic and CT Increased uptake in the maxilla, mandible, and skull on PET scan	16 months, no recurrence
6	Manolopoulos et al., 2003 [71]	38	M	Difficulties in swallowing for 2 months	Mass on the base of the tongue	Soft tissue mass on the base of the tongue that extended to the oropharynx and the proepiglottic space	17 months, no recurrence
7	Chan Lau et al., 2004 [72]	57	M	Chin numbness Lip ptosis Paroxysmal pain radiating from the preauricular region to the chin	Reduced pinprick sensation on the chin region, the scalp, and the brow	NA	12 months, no recurrence
8	Cascarini and Brown 2005 [73]	38	M	Painful mandibular swelling Ipsilateral numb lip	Mandibular vestibule swelling Lower lip numbness	No pathologic features	3 years, no recurrence
9	Nissenbaum et al., 2007 [74]	55	M	Non-healing extraction socket on the right posterior mandible Mandibular toothache for 5 weeks Lower lip numbness Right eyelid ptosis	Exophytic, firm mass on the right alveolar ridge overlying the extraction socket Palpable right submandibular lymph node	Ill-defined lytic lesion of the right body of the mandible	2 ½ years, no recurrence
10	Feinberg et al., 2007 [75]	80	M	Muffled voice, odynophagia, cough, and snoring for 3 months	Mass on the base of the tongue	Mass at the base of the tongue that crossed the midline and extended to the vallecula	2 ½ years, no recurrence
11	Balasubramaniam et al., 2009 [76]	36	F	Mandibular swelling Mandibular toothache Difficulty in mouth opening	Gingival erythema, ulcer, and suppuration Mandibular swelling	Periodontal ligament space widening Irregular bone loss on the mandibular alveolar ridge around the lesion	3 months, no recurrence
12	Martos-Diaz et al., 2009 [77]	29	M	Lower lip sensitive alteration for 1 month	Complete anesthesia of lower lip	No evidence of osteolysis Facial MRI showed a hypointense signal on T1 and hyperintense on T2 at the marrow level of the left mandibular angle and joint	3 years, no recurrence
13	Nikgoo et al., 2009 [78]	31	M	Extensive masses on maxilla at extraction sites Tooth mobility—extracted Chewing difficulty and bleeding from gums	Gingival bleeding Maxillary bilateral firm masses near the extraction sites Facial asymmetry	Periapical osteolysis Floating teeth Sinus bone destruction Space-occupying lesions within sinuses bilaterally	6 months, not satisfactory response
14	Keichiro et al., 2010 [79]	54	M	Numbness and slight chin pain Headache	Hypoesthesia below the lower lip on the 3rd division of the trigeminal nerve	Mandibular osteolysis	Died 11 months later
15	Sudhakara et al., 2011 [80]	42	M	Facial swelling for 1 year Tingling sensation in the same region	Palpable submandibular lymph node Facial asymmetry Solitary diffuse mass on the mandibular vestibule	No pathologic features	NA
16	Faltas et al., 2011 [81]	48	F	Numbness and tingling to the right side of the lower lip and chin for a few weeks	Hypoesthesia of the mental branch of the right trigeminal nerve	No pathologic features	2 years, no recurrence
17		37	M	Mandibular pain after routine dental cleaning	No pathologic signs	No pathologic features	5 years, intra-abdominal relapse No recurrence (after bone marrow transplantation)
18	García-Álvarez et al., 2012 [82]	58	M	Left chin paresthesia for 2 months Difficulty in swallowing and chewing Dysphonia	Reduced left facial sensation extending to the ipsilateral preauricular region Gag reflex absent Unilateral palatal palsy with the deviation of the uvula and the deviation of the tongue toward the left side with unilateral atrophy Sensitivity loss of the anterior two-thirds of the tongue	No pathologic features in the jaws	23 months, no recurrence
19	Kikutsi et al., 2012 [83]	61	F	Paresthesia of the lower lip	At first, dull pain in the left posterior mandible A few weeks later, rapid swelling of the left side of the face and buccal mucosa, trismus	Panoramic: No pathologic features CT: Extensive soft tissue mass in the left masseter and medial pterygoid muscle	11 months, no recurrence
20	Ho et al., 2013 [84]	59	M	Tooth mobility Mandibular/maxillary swelling Numbness sensation on the mandible for over 1 month	Mandibular/maxillary swelling Mandibular paresthesia Palpable neck lymph nodes	Mandibular/maxillary osteolysis	Died 1 month later
21	Barboza et al., 2013 [85]	35	M	Painless swelling in the maxilla for 2 months	Extraoral swelling in the left nasal wing and left upper canine area Intraoral: ulcerated, friable mass in the left portion of the maxilla, extending through the midline	Diffuse osteolytic lesion	6 months, no recurrence
22	Boffano et al., 2013 [86]	35	M	Bilateral swelling of the maxilla for 3 months	Ulcerative, nontender, soft swelling in the maxillary tuber bilaterally	NA	NA
23	Manne et al., 2014 [87]	21	M	Asymptomatic cheek swelling for 2 months Diplopia of the right eye	Intraoral firm, diffuse, and nontender swelling on the maxillary teeth-bearing area Extraoral mildly diffused, firm, and nontender swelling in the maxillary region Soft, tender, and non-adherent, palpable submandibular lymph node	Intact lamina dura with mild diffuse bony rarefactions No pathologic features on panoramic radiograph 4 weeks after an incisional biopsy of tumor mass involving the maxilla extending to the inferior wall of the orbit on CT	Died during chemotherapy
24	Rebelo-Pontes et al., 2014 [61]	22	M	Painful mass of the mandible	Painful exophytic mass on the alveolar ridge of the mandible	Osteolysis, root resorption, and the loss of lamina dura	1 year, no recurrence
25		54	F	Mandible swelling	Mandible swelling and tooth mobility	Osteolysis, root resorption, and the loss of lamina dura	2 years, no recurrence
26		19	M	Maxillary swelling	Exophytic mass on the alveolar ridge of the maxilla and tooth mobility	Osteolysis, root resorption, and the loss of lamina dura	Died of disease
27	Patankar et al., 2015 [88]	38	M	Painful swelling on the upper anterior gingiva	Swelling in mandibular and maxillary gingiva, tooth mobility, and displacement	No changes	Died of disease
28	Sethi et al., 2015 [89]	38	M	Swollen and bleeding gingiva (initially in the anterior maxillary region and gradually involving the whole maxillary and mandibular gingiva) for 6 months	Generalized enlargement and bleeding of the maxillary and mandibular gingiva Matted enlarged right submandibular lymph nodes Enlarged, mobile, and nontender cervical lymph nodes	NA	Lost to follow-up
29	Goto et al., 2016 [90]	27	F	Pain and numbness in the right lower premolar region and lower lip	Mandibular pain with numbness of the right lower lip	A lower density of bone trabeculae on the right side of the mandible without the erosion of the cortex	No recurrence
30	Garcia et al., 2017 [91]	42	M	Mild swelling of the mandible, lower lip paresthesia	Mandible swelling, tooth mobility	No changes	4 years, no recurrence
31	Kuo et al., 2017 [92]	29	M	Mandibular swelling	Two ulcerative gingival swellings at the buccal side of the left mandibular molar and retromolar area	Radiolucent lesion at the bifurcation and periapical areas of the left mandibular 1st molar	5 years, no recurrence
32	Pedraza et al., 2019 [93]	63	M	Bilateral painful mandibular swellings	Bilateral erythematous mandibular swellings affecting the posterior alveolar ridges, at the level of left premolars and right molars	NA	Died of disease
33	Tseng et al., 2021 [94]	84	M	Mandibular gingival swelling	Mandibular gingival swelling	Osteolysis in the mandible	Died of disease
34	Azimi et al., 2021 [19]	49	M	Pain in the left mandibular area and lower lip paresthesia	Mass with intact overlying mucosa and firm consistency at the left edentulous mandibular ridge	Ill-defined radiolucent lesion extending from the left alveolar crest to the inferior alveolar canal of the mandible with left buccal cortical plate invasion	3 months, no marrow involvement
35	Parker et al., 2021 [95]	37	F	Pain in the right side of the mandible and episodic numbness in the right lower lip and chin	Pain in the right side of the mandible and episodic numbness in the ipsilateral mental nerve distribution	Well-defined unilocular radiolucency at the apex of the right lower second molar	No recurrence
36	Stanbouly et al., 2022 [96]	37	F	Bilateral paresthesia of the lower lip and chin	At presentation: no clinical findings 1 month after the extractions of 3rd molars: soft tissue fungating from the socket of the mandibular 3rd molar, and mobility of the adjacent 2nd molar	No changes (panoramic radiograph) 1 month after the extractions of 3rd molars: vacuous trabecular spaces in the entirety of the mandible, and opacity in the sinus floor above the left maxillary 1st molar (on CT and panoramic radiograph)	6 months, no recurrence
37	Sodnom-Ish et al., 2022 [97]	31	M	Bilateral numbness and severe neurosensory disturbance in the lower lip and mandible	Mobility of the lower left 1st and 2nd molars	Generalized loss of lamina dura and periodontal ligament space widening of the posterior mandibular teeth (on CT and panoramic radiograph)	4 years, no recurrence
38	Tereshko et al., 2024 [98]	43	F	Numbness, paresthesia, and persistent burning pain involving both territories of the right and left mental nerves	Cervical lymph nodes swelling	Enlarged lymph nodes in the cervical region bilaterally	Undergoing treatment

## Data Availability

Department of Oral Medicine & Pathology and Hospital Dentistry, School of Dentistry, National and Kapodistrian University of Athens, Greece.
